# The effects of daily dose and treatment duration of metformin on the prevalence of vitamin B12 deficiency and peripheral neuropathy in Chinese patients with type 2 diabetes mellitus: A multicenter cross‐sectional study

**DOI:** 10.1111/1753-0407.13428

**Published:** 2023-06-13

**Authors:** Leili Gao, Xingwu Ran, Xuejun Liu, Xingping Shen, Shuchun Chen, Fuqiang Liu, Dong Zhao, Yan Bi, Qing Su, Yao Lu, Minxiang Lei, Yanfang Wang, Linong Ji

**Affiliations:** ^1^ Department of Endocrinology Peking University People's Hospital Beijing China; ^2^ Department of Endocrinology West China Hospital Sichuan University Chengdu China; ^3^ Department of Endocrinology Metabolic Disease Hospital of Tianjin Medical University Tianjin China; ^4^ Department of Endocrinology Zhongshan Hospital Xiamen University Xiamen China; ^5^ Department of Endocrinology Hebei General Hospital Shijiazhuang China; ^6^ Department of Endocrinology Qilu Hospital of Shangdong University Jinan China; ^7^ Department of Endocrinology Beijing Luhe Hospital Affiliated to Capital Medical University Beijing China; ^8^ Department of Endocrinology Nanjing Drum Tower Hospital Nanjing China; ^9^ Department of Endocrinology Xinhua Hospital Affiliated to Shanghai Jiaotong University School of Medicine Shanghai China; ^10^ Department of Endocrinology Second Affiliated Hospital of Xinjiang Medical University Xinjiang China; ^11^ Department of Endocrinology Xiangya Hospital Central South University Changsha China; ^12^ Department of Endocrinology Henan Provincial People's Hospital Zhengzhou China

**Keywords:** metformin, type 2 diabetes mellitus, vitamin B12 deficiency, 二甲双胍, 2型糖尿病, 维生素B12缺乏

## Abstract

**Aims:**

To evaluate the prevalence of vitamin B12 deficiency in Chinese patients with type 2 diabetes mellitus receiving metformin treatment and to investigate the effects of metformin daily dose and treatment duration on the prevalence of vitamin B12 deficiency and peripheral neuropathy (PN).

**Materials and Methods:**

In this multicenter cross‐sectional study, 1027 Chinese patients who had been taking ≥1000 mg/day metformin for ≥1 year were enrolled using proportionate stratified random sampling based on daily dose and treatment duration. Primary measures included the prevalence of vitamin B12 deficiency (<148 pmol/L), borderline B12 deficiency (148 pmol/L‐211 pmol/L), and PN.

**Results:**

The prevalence of vitamin B12 deficiency, borderline deficiency, and PN were 2.15%, 13.66%, and 11.59%, respectively. Patients receiving ≥1500 mg/day metformin had significantly higher prevalence of borderline vitamin B12 deficiency (16.76% vs. 9.91%, *p* = .0015) and serum B12 ≤221 pmol/L (19.25% vs. 11.64%, *p* < .001) than patients receiving <1500 mg/day metformin. No difference was found in prevalence of borderline vitamin B12 deficiency (12.58% vs. 15.49%, *p* = .1902) and serum B12 ≤221 pmol/L (14.91% vs. 17.32%, *p* = .3055) between patients receiving metformin for ≥3 and <3 years. Patients with vitamin B12 deficiency had numerically higher PN prevalence (18.18% vs. 11.27%, *p* = .3192) than patients without it. Multiple logistic analyses revealed that HbA1c and metformin daily dose were associated with the prevalence of borderline B12 deficiency and B12 ≤221 pmol/L.

**Conclusions:**

High daily dosage (≥1500 mg/day) played an important role in metformin‐associated vitamin B12 deficiency while not contributing to the risk of PN.

## Graphical abstract

**Highlights**

- This is the first multicenter, cross‐sectional study on the prevalence of vitamin B12 deficiency in Chinese patients with type 2 diabetes mellitus (T2DM) receiving metformin treatment.
- About one out of five patients with T2DM receiving ≥1500 mg/day metformin could develop vitamin B12 deficiency.
- High daily dosage of metformin (≥1500 mg/day) played an important role in metformin‐related vitamin B12 deficiency while not contributing to the risk of peripheral neuropathy.

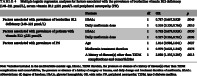

## 摘要

目的:了解中国接受二甲双胍治疗的2型糖尿病患者维生素B12缺乏症的患病率, 探讨二甲双胍日剂量和治疗时间对维生素B12缺乏症患病率和周围神经病变(PN)的影响。

方法:在这项多中心横断面研究中, 采用基于日剂量和治疗时间的比例分层随机抽样, 纳入了1027例服用二甲双胍≥1000 mg/d≥1年的中国患者。主要指标包括维生素B12缺乏症(<148 pmol/L), B12临界缺乏(148 pmol/L‐211 pmol/L)和PN的患病率。

结果:维生素B12缺乏, 临界缺乏和PN的患病率分别为2.15%, 13.66%和11.59%。接受≥1500mg /d二甲双胍治疗的患者与接受<1500mg /d二甲双胍治疗的患者相比, 维生素B12临界缺乏(16.76%vs.9.91%, p = 0.0015)和血清B12≤221 pmol/L(19.25%vs.11.64%, p<0.001)的患病率显著较高。服用二甲双胍≥3年和<3年的患者之间, 维生素B12临界缺乏(12.58%vs.15.49%, p = 0.1902)和血清B12≤221 pmol/L(14.91%vs.17.32%, p =0.3055)的患病率没有差异。与无维生素B12缺乏的患者相比, 缺乏维生素B12的患者PN患病率较高(18.18%vs.11.27%, p = 0.3192)。多元logistic回归分析显示, HbA_1c_和二甲双胍日剂量与B12临界缺乏和B12≤221 pmol/L的患病率相关。

结论:高日剂量(≥1500mg /天)在二甲双胍相关的维生素B12缺乏症中起重要作用, 但不会增加PN的风险。

## 1 INTRODUCTION

According to the 2022 International Diabetes Federation Diabetes Atlas, the prevalence of diabetes in China was 13.0%.^1^, ^2^ Metformin is the first‐line pharmacological treatment for type 2 diabetes mellitus (T2DM).^3^ Both the American Diabetes Association (ADA) Standards of Medical Care in Diabetes and the 2020 Chinese guideline on T2DM treatment recommend metformin as the preferred initial treatment and part of the combination therapy for T2DM unless contraindicated or intolerated.^4^, ^5^ However, it has been found that metformin treatment lowered patients' vitamin B12 level and caused biochemical vitamin B12 deficiency in some patients and that the prevalence of metformin‐related vitamin B12 deficiency ranged from 4.3% to 30%.^6^, ^7^, ^8^, ^9^, ^10^, ^11^ As vitamin B12 is a key cofactor in intracellular enzymatic reactions important in the functions of central nervous system and erythropoiesis^11^ and clinical manifestations of vitamin B12 deficiency include peripheral neuropathy (PN), cognitive impairment, macrocytic anemia and neuropsychiatric disorders,^12^, ^13^, ^14^ the ADA Standards of Medical Care in Diabetes recommended periodic vitamin B12 measurement in patients treated with metformin, especially in those with anemia or PN.^4^

Although it has been agreed by most that long‐term metformin treatment could decrease vitamin B12 level,^7^, ^8^, ^9^, ^10^, ^15^, ^16^, ^17^, ^18^, ^19^, ^20^, ^21^, ^22^, ^23^, ^24^, ^25^, ^26^ whether dose and/or treatment duration of metformin affect patients' vitamin B12 level remains controversial.^8^, ^9^, ^10^, ^15^, ^16^, ^17^, ^18^, ^19^, ^20^ Some studies found that high daily metformin dose was associated with decreased vitamin B12 level and increased risk of vitamin B12 deficiency,^8^, ^9^, ^17^ some found that it was metformin treatment duration that had such associations,^18^, ^20^ whereas other studies found that both metformin dose and treatment duration were associated with vitamin B12 level and risk of vitamin B12 deficiency.^10^, ^15^, ^16^, ^19^ Such inconsistent findings could be because the patients included in different studies received metformin treatment of different doses and/or different treatment duration, different cutoff points for metformin daily dose and/or treatment duration were used by different studies to evaluate their associations with vitamin B12 deficiency, and different standards of vitamin B12 deficiency and methods of statistics analyses were adopted by different studies. Additionally, studies on whether metformin‐related B12 deficiency led to clinical manifestations such as PN or anemia reached different conclusions.^3^, ^6^, ^9^, ^13^, ^16^, ^21^, ^22^ As metformin‐related PN could be mistaken for diabetic PN (DPN) and could irreversibly exacerbate DPN, vitamin B12 deficiency is reversible and treatable, and early detection of metformin‐related vitamin B12 deficiency is important.^11^, ^19^

Large‐scale multicenter studies on the prevalence of vitamin B12 deficiency in Chinese patients with T2DM taking metformin have been lacking. The current multicenter, cross‐sectional study aimed to make such evaluation as well as investigate the effects of daily dose and treatment duration of metformin on the prevalence of vitamin B12 deficiency and PN and on patients' hematological parameters using proportionate stratified random sampling to better reflect real‐life practice.

## 2 MATERIALS AND METHODS

This is a multicenter, cross‐sectional study conducted at 12 tertiary hospitals in China from 14 May 2020 to 28 January 2021. The study was approved by the independent ethics committee at each participating hospital and was conducted in accordance with the Declaration of Helsinki in 1995 (as revised in Fortaleza, Brazil, October 2013) and Good Clinical Practice guidelines of the Chinese National Medical Products Administration. All patients provided informed consent before screening and patient anonymity was preserved.

### 2.1 Patients

Patients aged 40–75 years with a body mass index of 19.0–35.0 kg/m^2^, diagnosed with T2DM according to the 1999 World Health Organization definition of T2DM (except glycated hemoglobin [HbA1c] ≥6.5% (48 mmol/mol) diagnostic criterion)^27^ who have been taking ≥1000 mg/day metformin for ≥1 year were screened and recruited. Exclusion criteria were described in the Appendix S1.

### 2.2 Sample size and sampling method

Although most previous studies reported that the prevalence of metformin‐related vitamin B12 deficiency ranged from 4.3% to 30%,^6^, ^7^, ^8^, ^9^, ^10^, ^11^ none of them was performed on Chinese patients. Ethnicity could affect the prevalence of metformin‐associated B12 deficiency.^20^ A study on Chinese institutionalized elder patients receiving metformin treatment revealed a 53.2% prevalence of B12 deficiency.^28^ As elder patients were at a greater risk of vitamin B12 deficiency,^27^ it is expected that younger patients would have lower prevalence. Another study on Chinese patients taking metformin found a 26.87% prevalence of B12 deficiency.^29^ A value in between, 35%, was chosen as the assumed prevalence of vitamin B12 deficiency in our study. Based on this assumption, 1025 patients provide a 2.9% margin of error at a 95% confidence interval. Proportionate stratified random sampling was used to enroll eligible patients from the 12 participating hospitals. Specifically, based on the real‐life proportion of patients taking ≥1000 and <1500 mg/day metformin for ≥1 and <3 years (Group A), taking ≥1500 mg/day metformin for ≥1 and <3 years (Group B), taking ≥1000 and <1500 mg/day metformin for ≥3 years (Group C), and taking ≥1500 mg/day metformin for ≥3 years (Group D), the number of patients enrolled in the four groups were determined, and when the designated number of patients were enrolled in a group, enrollment was terminated for that group. Additionally, patients within each group were enrolled using systematic random sampling (sampling interval = 3) at each participating hospital.

### 2.3 Data collection

Enrolled patients were asked to fast from 10 p.m. the night before the examination day (day 0). The following were measured: serum vitamin B12, serum homocysteine (Hcy), routine blood cell analysis (hemoglobin (Hb), mean corpuscular volume (MCV), and mean corpuscular hemoglobin (MCH)), HbA1c, and five tests for assessing PN (ankle reflex, vibration perception using a 128‐Hz tuning fork, 10‐g monofilament testing, pinprick, and temperature sensation).^30^, ^31^ Enrolled patients also completed the Michigan Neuropathy Screening Instrument and male patients completed the International Index of Erectile Function (IIEF‐5).

### 2.4 Aims

The primary aim was the prevalence of vitamin B12 deficiency (serum vitamin B12 <148 pmol/L), borderline B12 deficiency (148–211 pmol/L),^32^ and PN in the four groups of patients.

For patient with symptoms such as pain, numbness, or paresthesia, a diagnosis of PN was made with any one or more bilateral symmetrical abnormalities of the PN test[s]; For patients without these symptom[s], a diagnosis of PN was made with any two or more bilateral symmetrical abnormalities of the PN tests.

A bilateral symmetrical abnormal PN test was declared when both limbs of a patient had no response to or perception of the one of the following tests: ankle reflex, vibration perception using a 128‐Hz tuning fork, 10‐g monofilament testing, pinprick, and temperature sensation.

Secondary aims included:

1. The effects of daily dose and treatment duration of metformin on the prevalence of vitamin B12 deficiency, borderline B12 deficiency, and PN, as well as the prevalence of clinically meaningful abnormal HbA1c, Hb, MCV, and MCH;
2. Difference in the prevalence of clinically meaningful abnormal HbA1c, Hb, MCV, and MCH and the prevalence of PN among patients with vitamin B12 deficiency, borderline B12 deficiency, and normal B12 level;
3. Correlation between patients' serum vitamin B12 level and serum Hcy level;
4. Variables associated with the prevalence of vitamin B12 deficiency, borderline B12 deficiency, and PN; and.
5. The prevalence of suspected erectile dysfunction (ED) in male patients (IIEF‐5 ≤21indicated suspected ED^33^).

### 2.5 Statistical analysis

The SAS V9.4software (SAS Institute Inc.) was used for all statistical analyses in the study. The statistical analyses were performed on full analysis set (all enrolled patients; FAS). Data were expressed as *n* (%) for categorical variables and means ± SD for continuous variables. Comparisons between two groups were performed using the paired *t* test for normally distributed continuous data, the Wilcoxon rank sum test for nonnormally distributed continuous data, and the chi‐square test or Fisher's exact test for categorical data. The Kruskal–Wallis *H* test was used for comparisons among more than two groups. Significance of differences in the prevalence of vitamin B12 deficiency, borderline B12 deficiency, serum B12 ≤221 pmol/L, and PN among different groups was confirmed with Cochran–Mantel–Haenszel (CMH) chi‐square test adjusted for significant confounding factors including age (<65 and ≥65 years old), the presence of absence of clinically meaningful abnormal HbA1c as judged by the investigators based on its reference range (≤6.5%) and its clinical implications, a history of disease(s) other than T2DM complications and comorbidities, and a history of surgery or trauma (“adjusted CMH”). Correlation between the patients' serum vitamin B12 level and serum Hcy level was assessed using the Pearson correlation and the Spearman correlation. Taking center effect into consideration, multiple logistic analyses adjusted for metformin daily dose and treatment duration as well as other significant confounding factors were performed to identify variables associated with the prevalence of vitamin B12 deficiency, borderline B12 deficiency, vitamin B12 ≤221 pmol/L, and PN. All tests were two tailed and a *p* value <.05 indicated statistical significance.

## 3 RESULTS

### 3.1 Demographic and clinical characteristics

The study enrolled 1027 patients. Among them, 208, 174, 257, and 388 patients were in Groups A, B, C, and D, respectively (Table 1). Except for age, heart rate, T2DM duration, HbA1c, history of diseases others than T2DM complications and comorbidities, and history of surgery/trauma, the four groups had comparable demographic and clinical characteristics (Table 1).

**TABLE 1:** Demographic and clinical characteristics of enrolled patients (FAS).

Variable	Group A (Metformin ≥1000 and <1500 mg/day for ≥1 and <3 years; *N* = 208)	Group B (Metformin ≥1500 mg/day for ≥1 and <3 years; *N* = 174)	Group C (Metformin ≥1000 and <1500 mg/day for ≥3 years; *N* = 257)	Group D (Metformin ≥1500 mg/day for ≥3 years; *N* = 388)	*p* value*	Adjusted CMH *p* value**
Age (years)	58.27 ± 7.93	56.30 ± 8.72	60.38 ± 8.39	59.58 ± 8.04	<.0001	–
Male, *n* (%)	107 (51.4%)	99 (56.9)	135 (52.5%)	199 (51.3%)	.6441	–
Height (cm)	165.09 ± 8.70	164.47 ± 8.51	164.25 ± 8.71	164.13 ± 8.47	.61	–
Weight (kg)	70.54 ± 12.11	69.73 ± 10.85	69.29 ± 12.58	69.91 ± 11.01	.7174	–
SBP (mmHg)	131.84 ± 14.11	130.14 ± 13.85	132.79 ± 14.50	132.92 ± 14.63	.1643	–
DBP (mmHg)	80.37 ± 8.80	80.41 ± 9.20	80.37 ± 9.62	79.87 ± 9.50	.8702	–
Heart rate (beats)	76.94 ± 9.64	78.53 ± 10.13	76.49 ± 8.93	78.28 ± 9.86	.0484	–
T2DM duration (years)	5.09 ± 4.82	5.34 ± 5.31	9.37 ± 6.38	10.87 ± 6.06	<.001	–
BMI (kg/m^2^)	25.79 ± 3.44	25.71 ± 3.03	25.56 ± 3.29	25.89 ± 3.17	.6494	–
Medical history—T2DM complications and comorbidities						
Retinopathy, *n* (%)	11 (5.3%)	8 (4.6%)	23 (8.9%)	18 (4.6%)	.268	–
Nephropathy, *n* (%)	6 (2.9%)	0 (0%)	6 (2.33%)	8 (2.1%)	.3311	–
CVDs, *n* (%)	7 (3.4%)	6 (3.4%)	19 (7.4%)	24 (6.2%)	.4718	–
Cerebrovascular diseases, *n* (%)	4 (1.9%)	1 (0.6%)	8 (3.1%)	5 (1.5%)	.3521	–
PVDs, *n* (%)	4 (1.9%)	1 (0.6%)	7 (2.7%)	4 (1.0%)	.2942	–
PN, *n* (%)	10 (4.8%)	8 (4.6%)	19 (7.4%)	22 (5.7%)	.7201	–
Medical history—other diseases, *n* (%)	97 (46.6%)	100 (57.5%)	149 (58.0%)	245 (63.1%)	<.0017	–
Medical history—surgery/trauma, *n* (%)	67 (32.2%)	41 (23.6%)	77 (30.0%)	145 (37.4%)	.0120	–
Taking vitamin supplements, *n* (%)	0 (0%)	1 (0.6%)	0 (0%)	4 (1.0%)	.1973	–
HbA1c (%)	6.99 ± 1.24	7.12 ± 1.21	7.44 ± 1.34	7.51 ± 1.36	<.0001	–
Serum vitamin B12 status						
Definite vitamin B12 deficiency (<148 pmol/L), *n* (%)	2 (1.0%)	5 (2.9%)	6 (2.3%)	9 (2.3%)	.5831	0.5511
Borderline B12 deficiency (148 pmol/L ≤ B12 ≤ 221 pmol/L), *n* (%)	23 (11.1%)	36 (20.8%)	23 (9.0%)	58 (14.9%)	.0029	0.0033
Patients with serum B12 ≤ 221 pmol/L, *n* (%)	25 (12.0%)	41 (23.7%)	29 (11.3%)	67 (17.3%)	.002	0.0016
Serum B12 level (pmol/L)	418.90 ± 251.21	359.82 ± 190.63	442.66 ± 249.18	378.92 ± 218.95	<.001	–
PN, *n* (%)	12 (5.8%)	26 (14.9%)	33 (12.8%)	48 (12.4%)	.0243	0.0392
Possible ED (IIEF‐5 ≤ 21) in male patients, *n* (%)	82/106 (77.4%)	84/99 (84.8%)	119/135 (88.1%)	163/199 (81.9)	.1474	–

*Note*: Data are expressed as means ± SDs for continuous variables and *n* (%) for categorical variables.

Abbreviations: BMI, body mass index; CMH, Cochran–Mantel–Haenszel test; CVDs, cardiovascular disease; DBP, diastolic blood pressure; ED, erectile dysfunction; FAS, full analysis set; HbA1c, glycated hemoglobin; IIEF‐5, International Index of Erectile Function; PN, peripheral neuropathy; PVDs, peripheral vascular diseases; SBP, systolic blood pressure; T2DM, type 2 diabetes mellitus.

* *p* value for Kruskal‐Wallis *H* test.

** *p* value for Cochran–Mantel–Haenszel (CMH) chi‐square test adjusted for significant confounding factors including age (<65 and ≥65 years old), the presence of absence of clinically meaningful abnormal HbA1c judged by the investigators based on its reference range (≤6.5%) and its clinical implications, the presence or absence of a history of disease(s) other than T2DM complications and comorbidities, and the presence of absence of a history of surgery or trauma.

### 3.2 The prevalence of vitamin B12 deficiency, borderline B12 deficiency, serum B12 ≤221 pmol/L, and PN

The prevalence of vitamin B12 deficiency was 2.15% (22/1025), and the prevalence of borderline deficiency and serum B12 ≤221 pmol/L was 13.66% (140/1025) and 15.80% (162/1025), respectively. Finally, the prevalence of PN was 11.59% (119/1027).

The four groups of patients had comparable prevalence of vitamin B12 deficiency. However, Group B had the highest prevalence of borderline B12 deficiency and serum B12 ≤221 pmol/L, with Group C having the lowest ones (Table 1), and serum B12 level was lowest in Group B and highest in Group C (Table 1).

Finally, the prevalence of PN was lowest in Group A and highest in Group B (Table 1).

### 3.3 Patients receiving ≥1500 mg/day metformin had significantly higher prevalence of borderline B12 deficiency and serum B12 ≤221 pmol/L as well as significantly lower serum B12 level than patients receiving ≥1000 and <1500 mg/day metformin

Comparable prevalence of vitamin B12 deficiency was found between patients receiving ≥1000 and <1500 mg/day metformin (Groups A + C) and patients receiving ≥1500 mg/day metformin (Groups B + D; 1.72% vs. 2.50%, *p* = .3963, adjusted CMH *p* = 0.3203). However, patients receiving ≥1500 mg/day metformin had significantly higher prevalence of borderline B12 deficiency (16.73% vs. 9.90%, *p* =0.0015, adjusted CMH *p* =.004) and serum B12 ≤221 pmol/L (19.25% vs. 11.64%, *p* < .001, adjusted CMH *p* = 0.002) than those receiving ≥1000 and <1500 mg/day metformin. Furthermore, patients receiving ≥1500 mg/day metformin had significantly lower serum vitamin B12 level (373.03 ± 210.64 pmol/L vs. 432.01 ± 250.10 pmol/L, *p* < .001; Table 2).

**TABLE 2:** The effect of daily dose and treatment duration of metformin on patients' serum vitamin B12 level, HbA1c, hematological parameters and on male patients' erectile function.

Variables	Metformin treatment ≥1000 and <1500 mg/day (*N* = 465)	Metformin treatment ≥1500 mg/day (*N* = 562)	*p* value
Serum B12 level (pmol/L)	432.01 ± 250.10	373.03 ± 210.64	<.001
B12 deficiency (<148 pmol/L), *n* (%)	8 (1.72%)	14 (2.50)	.3963
Borderline B12 deficiency (148 pmol/L ≤ B12 ≤ 221 pmol/L), *n* (%)	46 (9.90%)	94 (16.73%)	.0015
HbA1c (%)	7.25 ± 1.31	7.38 ± 1.33	.132
Clinically meaningful abnormal HbA1c^a^, *n* (%)	368 (79.1%)	449 (79.9%)	.7657
Clinically meaningful abnormal Hb^a^, *n* (%)	4 (0.9%)	4 (0.7%)	.7872
Clinically meaningful abnormal MCV^a^, *n* (%)	4 (0.9%)	2 (0.4%)	.2909
Clinically meaningful abnormal MCH^a^, *n* (%)	2 (0.4%)	1 (0.2%)	.4559
Suspected ED (IIEF‐5 ≤21) in male patients, *n* (%)	201/241 (83.4%)	247/298 (82.9%)	.8735

	Metformin treatment for ≥1 and <3 years (*N* = 381)	Metformin treatment for ≥3 years (*N* = 644)	
Serum B12 level (pmol/L)	392.07 ± 227.36	404.26 ± 233.34	.3727
B12 deficiency (<148 pmol/L), *n* (%)	7 (1.84%)	15 (2.33%)	.5995
Borderline B12 deficiency (148 pmol/L ≤ B12 ≤ 221 pmol/L), *n* (%)	59 (15.49%)	81 (12.58%)	.1902
HbA1c (%)	7.05 ± 1.23	7.48 ± 1.35	<.001
Clinically meaningful abnormal HbA1c^a^, *n* (%)	292 (76.44%)	525 (81.40%)	.057
Clinically meaningful abnormal Hb^a^, *n* (%)	4 (1.05%)	4 (0.62%)	.451
Clinically meaningful abnormal MCV^a^, *n* (%)	3 (0.79%)	3 (0.47%)	.5134
Clinically meaningful abnormal MCH^a^, *n* (%)	2 (0.52%)	1 (0.16%)	.2897
Suspected ED (IIEF‐5 ≤ 21) in male patients, *n* (%)	166/205 (80.98%)	282/334 (84.43%)	.2985

*Note*: Data are expressed as means ± SDs for continuous variables and *n* (%) for categorical variables.

Abbreviations: ED, erectile dysfunction; Hb, hemoglobin; HbA1c, glycated hemoglobin; IIEF‐5, International Index of Erectile Function; MCH, mean corpuscular hemoglobin; MCV, mean corpuscular volume.

^a^ Clinically meaningful abnormal HbA1c, Hb, MCV, and MCH were judged by the investigators based on their reference ranges (HbA1c ≤6.5%, Hb 120–160 g/L [men] and 110–150 g/L [women], MCV 80–100 fl, and MCH 27–34 pg) and their clinical implications.

Meanwhile, patients taking metformin for ≥1 and <3 years (Groups A + B) and patients taking metformin for ≥3 years (Groups C + D) had comparable prevalence of vitamin B12 deficiency, borderline B12 deficiency and serum B12 ≤221 pmol/L as well as comparable serum vitamin B12 level (Table 2; all *p* and adjusted CMH *p* > .05).

### 3.4 Patients receiving metformin treatment for ≥3 years had numerically higher prevalence of PN than patients receiving metformin treatment for ≥1 and <3 years and patients with vitamin B12 deficiency had numerically higher prevalence of PN than patients with serum B12 ≥148 pmol/L but without significance

Patients receiving metformin ≥1000 and <1500 mg/day metformin and patients receiving ≥1500 mg/day metformin had numerically higher prevalence of PN (9.68% vs. 13.17%, *p* = .082, adjusted CMH *p* = .1199) but without significance. Meanwhile, patients taking metformin for ≥3 years had numerically higher prevalence of PN than patients taking metformin for ≥1 and <3 years (12.56% vs. 9.95%, *p* = .2065, adjusted CMH *p* = .5205) but without significance.

Additionally, patients with vitamin B12 deficiency had numerically higher prevalence of PN than patients with serum B12 ≥148 pmol/L (18.18% vs. 11.27%, *p* = .3129) but without significance.

### 3.5 Longer treatment duration was associated with significantly higher HbA1c level, whereas patients' hematological parameters were not affected by metformin daily dose, treatment duration, or their vitamin B12 status

The overall prevalence of clinically meaningful abnormal HbA1c was high (817/1024 [79.79%]), and the overall prevalence of clinically meaningful abnormal Hb (8/1025 [0.78%]), MCV (6/1025 [0.59%]), and MCH (3/1025 [0.29%]) as judged by the investigators was very low (Table 2).

Patients in the two dosage groups had comparable HbA1c level and comparable prevalence of clinically meaningful abnormal HbA1c, Hb, MCV, and MCH as judged by the investigators (*p* all >.05; Table 2).

On the other hand, although the prevalence of clinically meaningful abnormal HbA1c, Hb, MCV, and MCH as judged by the investigators were comparable between patients receiving metformin treatment for ≥1 and <3 years and patients receiving metformin treatment for ≥3 years (*p* all >.05), patients taking metformin for ≥3 years had significantly higher HbA1c level (7.48 ± 1.35% vs. 7.05 ± 1.23%, *p* < .001; Table 2).

Finally, patients with different vitamin B12 status had comparable prevalence of clinically meaningful abnormal HbA1c, Hb, MCV, and MCH (*p* all >.05; Table 3).

**TABLE 3:** Comparison of patients' HbA1c, hematological parameters and prevalence of PN according to their vitamin B12 status.

Variables	Definite vitamin B12 deficiency (B12 < 148 pmol/L; *N* = 22)	Borderline vitamin B12 deficiency (148 pmol/L ≤ B12 ≤ 221 pmol/L; *N* = 140)	Normal vitamin B12 level (>221 pmol/L; *N* = 853)	*p* value
HbA1c (%)	7.21 ± 1.13	7.03 ± 1.01	7.37 ± 1.37	.0155
Suspected ED (IIEF‐5 ≤ 21) in male patients, *n* (%)	11/11 (100%)	65/73 (89.04%)	370/453 (81.68%)	.0947
PN *n* (%)	4 (18.18%)	18 (12.86%)	95 (11.01%)	.4903
Clinically meaningful abnormal HbA1c^a^, *n* (%)	15 (68.18%)	111 (79.29%)	683 (80.16%)	.4814
Clinically meaningful abnormal Hb^a^, *n* (%)	0 (0%)	2 (1.43%)	6 (0.70%)	.7513
Clinically meaningful abnormal MCV^a^, *n* (%)	0 (0%)	1 (0.71%)	5 (0.59%)	.4681
Clinically meaningful abnormal MCH^a^, *n* (%)	0 (0%)	0 (0%)	3 (0.35%)	.4509

Abbreviations: ED, erectile dysfunction; Hb, hemoglobin, HbA1c, glycated hemoglobin; IIEF‐5, International Index of Erectile Function; MCH, mean corpuscular hemoglobin; MCV, mean corpuscular volume; PN, peripheral neuropathy.

^a^ Clinically meaningful abnormal HbA1c, Hb, MCV, and MCH were judged by the investigators based on their reference ranges (HbA1c ≤6.5%, Hb 120–160 g/L [men] and 110–150 g/L [women], MCV 80–100 fl 4, and MCH 27–34 pg) and their clinical implications.

### 3.6 Patients' serum vitamin B12 level had negative correlation with their serum Hcy level

The Pearson correlation and the Spearman correlation revealed a negative correlation between the patients' serum vitamin B12 level and their serum Hcy level (coefficient = −0.25655, *p* < .001; and coefficient = −0.31967, *p* < .0001, respectively).

### 3.7 Multiple logistic analyses revealed that HbA1c and daily dose of metformin were associated with the prevalence of borderline B12 deficiency and B12 ≤221 pmol/L

As there were only 22 patients with vitamin B12 deficiency, a number too small for a proper multiple logistic analysis, we did not perform one to identify factor associated with the prevalence of vitamin B12 deficiency.

According to multiple logistic analyses adjusted for age, HbA1c, T2DM duration, the presence or absence of a history of disease(s) other than T2DM complications and comorbidities, the presence or absence of a history of surgery or trauma, and daily dosage and treatment duration of metformin, HbA1c and daily dose of metformin were associated with the prevalence of borderline B12 deficiency and B12 ≤221 pmol/L (Table 4), The higher the HbA1c, the lower the prevalence of borderline B12 deficiency and B12 ≤221 pmol (odds ratio [OR] 0.787, *p* = .0045; and OR 0.792, *p* = .0030, respectively). Meanwhile, the lower the daily dose of metformin, the lower the prevalence of borderline B12 deficiency and B12 ≤221 pmol (OR 0.766, *p* = .0010; and OR 0.527, *p* = .0006, respectively; Table 4). There was no interaction between metformin daily dose and treatment duration in any of the models (*p* all >.05).

**TABLE 4:** Multiple logistic regression analyses for factors associated with the prevalence of borderline vitamin B12 deficiency (148–221 pmol/L), serum vitamin B12 ≤221 pmol/L and peripheral neuropathy (PN).

	Factors	df	OR	*p*
Factors associated with prevalence of borderline B12 deficiency (148–221 pmol/L)	HbA1c	1	0.787 (0.667,0.929	.0045
	Daily metformin dose	1	0.518 (0.350,0.766)	.0010
Factors associated with prevalence of patients with vitamin B12 ≤221 pmol/L	HbA1c	1	0.792 (0.679,0.924)	.0030
	Daily metformin dose	1	0.527 (0.366,0.761)	.0006
Factors associated with prevalence of PN	Age	1	1.014 (0.988,1.041)	.3027
	Metformin treatment duration	1	0.979 (0.623,1.540)	.9275
	A history of disease(s) other than T2DM complications and comorbidities	1	2.098 (1.335,3.295)	.0013

*Note*: Variables included in the multivariable models: age, HbA1c, T2DM duration, the presence or absence of a history of disease(s) other than T2DM complications and comorbidities, the presence or absence of a history of surgery or trauma, and daily dosage and treatment duration of metformin, HbA1c.

Abbreviations: df, degree of freedom; HbA1c, glycated hemoglobin; OR, odds ratio; PN, peripheral neuropathy; T2DM, type 2 diabetes mellitus.

### 3.8 The prevalence of suspected ED (IIEF‐5 ≤21) in male patients was very high

The prevalence of suspected ED in male patients was 83.12% (448/539). Difference between treatment duration and daily dose of metformin had no effect on the prevalence of suspected ED in the male patients.

## 4 DISCUSSION

In this first multicenter, cross‐sectional study on the prevalence of vitamin B12 deficiency in Chinese patients with T2DM receiving metformin treatment, we found that among Chinese patients aged 40–75 years who had been taking ≥1000 mg/day metformin for ≥1 year, the prevalence of vitamin B12 deficiency, borderline deficiency, serum B12 ≤221 pmol/L and PN was 2.14%, 13.63%, 15.77% and 25.90%, respectively. Patients receiving ≥1500 mg/day metformin had significantly higher prevalence of borderline B12 deficiency and B12 ≤221 pmol/L and lower serum vitamin B12 level than patients receiving <1500 mg/day metformin. Patients with vitamin B12 deficiency had only numerically higher PN prevalence (18.18% vs. 11.27%, *p* = .3192) than patients without it. No difference was found in prevalence of borderline vitamin B12 deficiency (12.58% vs. 15.49%, *p* = .1902) and serum B12 ≤221 pmol/L (14.91% vs. 17.32%, *p* = .3055) between patients receiving metformin for ≥3 and <3 years. According to multiple logistic analysis adjusted for significant confounding factors, HbA1c level and daily dose of metformin were associated with the prevalence of borderline B12 deficiency and B12 ≤221 pmol/L.

Previous studies have found that the prevalence of metformin‐associated vitamin B12 deficiency could range from 4.3% to 30%.^6^, ^7^, ^8^, ^9^, ^10^, ^11^ The prevalence of vitamin B12 deficiency (2.15%) and borderline deficiency (13.66%) in our study were a little lower than those reported by Aroda et al (4.3% and 19.1%, respectively)^7^ and Reinslatler et al (5.8% and 16.2%, respectively)^30^; this could be because our study excluded vegetarians, a group at high risk of B12 deficiecy.^34^, ^35^

Whether metformin dose, treatment duration, or both affected patients' vitamin B12 level has been under debate.^7^, ^8^, ^9^, ^10^, ^15^, ^16^, ^17^, ^18^, ^19^, ^20^ Our study indicated that it was the daily dose of metformin that affected a patient's vitamin B12 level. Although our study found no difference in the prevalence of vitamin B12 deficiency between the two metformin dosage groups, it could be because the number of patients with vitamin B12 deficiency^22^ in our study was too small. We especially concur with Kim et al.^17^ who concluded, “Metformin at ≥1500mg/d could be a major factor related to vitamin B12 deficiency.”

Besides metformin daily dose, multiple logistic analysis found HbA1c level to be negatively associated with the prevalence of borderline vitamin B12 deficiency and serum B12 ≤221 pmol/L. Both Ahmed et al and Kang et al had similar findings.^3^, ^36^ However, as a person's HbA1c level could be affected by many factors, the mechanism underlying such association and its clinical implication are currently not clear.

The clinical implications of metformin‐related vitamin B12 deficiency as to whether it could lead to manifestations such as PN or anemia have also been under debate.^3^, ^6^, ^9^, ^13^, ^16^, ^21^, ^22^, ^23^, ^24^ This controversy could be because body store of vitamin B12 is enormous relative to daily B12 consumption, and it could take up to 5 years for symptoms of vitamin B12 deficiency to occur.^11^, ^13^ We found that the prevalence of PN was not associated with metformin dose or treatment duration. Our observation was consistent with Russo et al who found that the lack of vitamin B12 secondary to the use of metformin did not significantly increase the frequency of peripheral neuropathy.^9^, ^14^, ^22^ As we found that metformin daily dose was associated with vitamin B12 deficiency, whereas PN prevalence was not associated with metformin daily dose or treatment duration, some would consider the possibility that PN was not directly associated with metformin‐associated B12 deficiency. Our findings are also in concordance with the results of the cross‐sectional study of Chen et al, which revealed no significant differences between metformin users and nonusers when neuropathy status was assessed by both objective (monofilament and neurothesiometry) and relatively subjective (questionnaires) measures.^3^, ^23^ A possible explanation for our findings was that the neuropathic effect caused by metformin‐related vitamin B12 deficiency could at least be partially counteracted by metformin's neuroprotective effect during early years of metformin use.^3^, ^11^, ^37^ Metformin exerts its neuroprotective effect through its glucose lowering action and it antihyperglycemic‐independent function such as inhibiting oxidative stress induced neuronal apoptosis.^3^, ^11^, ^37^

Based on our findings that about one out of five patients with T2DM receiving ≥1500 mg/day metformin could develop vitamin B12 deficiency or borderline deficiency, we think it is reasonable to consider periodic vitamin B12 measurement in patients treated with metformin, as recommended by the ADA Standards of Medical Care in Diabetes,^4^ especially in patients receiving ≥1500 mg/day metformin.

Our study was limited by the fact that it is a cross‐sectional study, therefore our results demonstrate associations rather than causal relationships. Also we screened and recruited patients with T2DM basing on 1999 World Health Organization diagnostic criteria except HbA1c because the HbA1c test method being used before lacksevidence that it is NGSP certified and standardized to the DCCT assay. It is a potential limitation. Furthermore, we did not explicitly exclude patients who used proton pump inhibitors and/or histamine H2 blockers, both being reportedly associated with vitamin B12 deficiency.^18^ On the other hand, our study used proportionate stratified random sampling based on daily dose and treatment duration of metformin to reflect real‐life practice; therefore, our study has improved sample precision with reduced sampling error.

In conclusion, high daily dosage of metformin (≥1500 mg/day) played an important role in metformin‐related vitamin B12 deficiency.

## AUTHOR CONTRIBUTIONS

Linong Ji contributed substantially to the conception and design of the work. Linong Ji and Leili Gao contributed substantially to acquisition, analysis, interpretation of data and wrote the first draft of the work; All authors contributed to the manuscript or revising it critically for important intellectual content; All authors gave final approval to the version to be published and agreed to be accountable for all aspects of the work in ensuring that questions related to the accuracy or integrity of any part of the work are appropriately investigated and resolved.

## FUNDING INFORMATION

All authors received research grants for this study from Eisai China Inc. in accordance with Good Publication Practice (GPP) guidelines (www.ismpp.org/gpp3).

## Supporting information


Appendix S1.

## References

[1] International Diabetes Federation . IDF Diabetes Atlas. 10th ed. International Diabetes Federation; 2021. Accessed 2022. Available from: https://www.diabetesatlas.org/

[2] Xu Y , Wang L , He J , et al. Prevalence and control of diabetes in Chinese adults. JAMA. 2013;310:948‐959. 24002281 10.1001/jama.2013.168118

[3] Ahmed MA , Muntingh G , Rheeder P . Vitamin B12 deficiency in metformin‐treated type‐2 diabetes patients, prevalence and association with peripheral neuropathy. BMC Pharmacol Toxicol. 2016;17:44. 27716423 10.1186/s40360-016-0088-3 PMC5054613

[4] American Diabetes Association . 9. Pharmacologic approaches to glycemic treatment: standards of medical Care in Diabetes‐2023. Diabetes Care. 2023;46:S140‐S157. 31862752 10.2337/dc20-S009

[5] Chinese Diabetes Society . Guidelines for the prevention and control of type 2 diabetes in China (2020 edition). Chin J Diabetes Mellit. 2021;13:315‐409. (Chinese).

[6] Singh AK , Kumar A , Karmakar D , Jha RK . Association of B12 deficiency and clinical neuropathy with metformin use in type 2 diabetes patients. J Postgrad Med. 2013;59:253‐257. 24346380 10.4103/0022-3859.123143

[7] Aroda VR , Edelstein SL , Goldberg RB , et al. Long‐term metformin use and vitamin B12 deficiency in the diabetes prevention program outcomes study. J Clin Endocrinol Metab. 2016;101:1754‐1761. 26900641 10.1210/jc.2015-3754 PMC4880159

[8] Beulens JW , Hart HE , Kuijs R , et al. Influence of duration and dose of metformin on cobalamin deficiency in type 2 diabetes patients using metformin. Acta Diabetol. 2015;52:47‐53. 24908579 10.1007/s00592-014-0597-8

[9] de Groot‐Kamphuis DM , van Dijk PR , Groenier KH , Houweling ST , Bilo HJ , Kleefstra N . Vitamin B12 deficiency and the lack of its consequences in type 2 diabetes patients using metformin. Neth J Med. 2013;71:386‐390. 24038568

[10] Ting RZ , Szeto CC , Chan MH , Ma KK , Chow KM . Risk factors of vitamin B(12) deficiency in patients receiving metformin. Arch Intern Med. 2006;166:1975‐1979. 17030830 10.1001/archinte.166.18.1975

[11] Yang W , Cai X , Wu H , Ji L . Associations between metformin use and vitamin B12 levels, anemia, and neuropathy in patients with diabetes: a meta‐analysis. J Diabetes. 2019;11:729‐743. 30615306 10.1111/1753-0407.12900

[12] Roy RP , Ghosh K , Ghosh M , et al. Study of vitamin B12 deficiency and peripheral neuropathy in metformin‐treated early type 2 diabetes mellitus. Indian J Endocrinol Metab. 2016;20:631‐637. 27730072 10.4103/2230-8210.190542 PMC5040042

[13] Hendrawati YD , Andrajati R , Supardi S , Ariyani A . The risk of cobalamin deficiency symptoms related to long‐term metformin use in T2DM patients. Acta Endocrinol. 2018;14:49‐54. 10.4183/aeb.2018.49 PMC6516611 31149236

[14] Russo GT , Giandalia A , Romeo EL , et al. Diabetic neuropathy is not associated with homocysteine, folate, vitamin B12 levels, and MTHFR C677T mutation in type 2 diabetic outpatients taking metformin. J Endocrinol Invest. 2016;39:305‐314. 26233336 10.1007/s40618-015-0365-9

[15] Haeusler S , Parry‐Strong A , Krebs JD . The prevalence of low vitamin B12 status in people with type 2 diabetes receiving metformin therapy in New Zealand—a clinical audit. N Z Med. 2014;127:8‐16. 25331307

[16] Hashem MM , Esmael A , Nassar AK , el‐Sherif M . The relationship between exacerbated diabetic peripheral neuropathy and metformin treatment in type 2 diabetes mellitus. Sci Rep. 2021;11:1940. 33479439 10.1038/s41598-021-81631-8 PMC7820469

[17] Kim J , Ahn CW , Fang S , Lee HS , Park JS . Association between metformin dose and vitamin B12 deficiency in patients with type 2 diabetes. Medicine. 2019;98:e17918. 31725641 10.1097/MD.0000000000017918 PMC6867725

[18] Damião CP , Rodrigues AO , Pinheiro MF , et al. Prevalence of vitamin B12 deficiency in type 2 diabetic patients using metformin: a cross‐sectional study. Sao Paulo Med J. 2016;134:473‐479. 28076635 10.1590/1516-3180.2015.01382111 PMC11448733

[19] Ko SH , Ko SH , Ahn YB , et al. Association of vitamin B12 deficiency and metformin use in patients with type 2 diabetes. J Korean Med Sci. 2014;29:965‐972. 25045229 10.3346/jkms.2014.29.7.965 PMC4101785

[20] Krishnan GD , Zakaria MH , Yahaya N . Prevalence of vitamin B12 deficiency and its associated factors among patients with type 2 diabetes mellitus on metformin from a district in Malaysia. J ASEAN Fed Endocr Soc. 2020;35:163‐168. 33442187 10.15605/jafes.035.02.03 PMC7784158

[21] Gupta K , Jain A , Rohatgi A . An observational study of vitamin b12 levels and peripheral neuropathy profile in patients of diabetes mellitus on metformin therapy. Diabetes Metab Syndr. 2018;12:51‐58. 28882470 10.1016/j.dsx.2017.08.014

[22] Olt S , Oznas O . Investigation of the vitamin B12 deficiency with peripheral neuropathy in patients with type 2 diabetes mellitus treated using metformin. North Clin Istanb. 2017;4:233‐236. 29270571 10.14744/nci.2017.98705 PMC5724917

[23] Chen S , Lansdown AJ , Moat SJ , et al. An observational study of the effect of metformin on B12 status and peripheral neuropathy. Br J Diabetes Vascular Dis. 2012;12:189‐193.

[24] Sato Y , Ouchi K , Funase Y , Yamauchi K , Aizawa T . Relationship between metformin use, vitamin B12 deficiency, hyperhomocysteinemia and vascular complications in patients with type 2 diabetes. Endocr J. 2013;60:1275‐1280. 24018893 10.1507/endocrj.ej13-0332

[25] Hoogeveen EK , Kostense PJ , Jakobs C , Bouter LM , Heine RJ , Stehouwer CDA . Does metformin increase the serum total homocysteine level in non‐insulin‐dependent diabetes mellitus? J Intern Med. 1997;242:389‐394. 9408068 10.1046/j.1365-2796.1997.00231.x

[26] Sahin M , Tutuncu NB , Ertugrul D , Tanaci N , Guvener ND . Effects of metformin or rosiglitazone on serum concentrations of homocysteine, folate, and vitamin B12 in patients with type 2 diabetes mellitus. J Diabetes Complications. 2007;21:118‐123. 17331860 10.1016/j.jdiacomp.2005.10.005

[27] World Health Organization . Definition, Diagnosis and Classification of Diabetes Mellitus and Its Complications: Report of a WHO Consultation. Part 1, Diagnosis and Classification of Diabetes Mellitus. World Health Organization; 1999 Accessed: November 2019. Available from https://apps.who.int/iris/handle/10665/66040

[28] Wong CW , Leung CS , Leung CP , Cheng JN . Association of metformin use with vitamin B12 deficiency in the institutionalized elderly. Arch Gerontol Geriatr. 2018;79:57‐62. 30114554 10.1016/j.archger.2018.07.019

[29] Cai Y , Shen J . Process of vitamin B12 deficiency in patients with type 2 diabetes mellitus induced by metformin. Eval Anal Drug‐Use Hospitals China. 2021;21:1021‐1024.

[30] Kamenov ZA , Petrova JJ , Christov VG . Diagnosis of diabetic neuropathy using simple somatic and a new autonomic (neuropad) tests in the clinical practice. Exp Clin Endocrinol Diabetes. 2010;118:226‐233. 20200815 10.1055/s-0030-1247565

[31] Paisley A , Abbott C , van Schie C , Boulton A . A comparison of the Neuropen against standard quantitative sensory‐threshold measures for assessing peripheral nerve function. Diabet Med. 2002;19:400‐405. 12027928 10.1046/j.1464-5491.2002.00706.x

[32] Reinstatler L , Qi YP , Williamson RS , Garn JV , Oakley GP Jr . Association of biochemical B_12_ deficiency with metformin therapy and vitamin B_12_ supplements: the National Health and nutrition examination survey, 1999‐2006. Diabetes Care. 2012;35:327‐333. 22179958 10.2337/dc11-1582 PMC3263877

[33] Xu Y , Zhang Y , Yang Y , Liu L , Chen Y , Liu X . Prevalence and correlates of erectile dysfunction in type 2 diabetic men: a population‐based cross‐sectional study in Chinese men. Int J Impot Res. 2019;31:9‐14. 30104671 10.1038/s41443-018-0060-4

[34] Oussalah A , Levy J , Berthezène C , Alpers DH , Guéant JL . Health outcomes associated with vegetarian diets: an umbrella review of systematic reviews and meta‐analyses. Clin Nutr. 2020;39:3283‐3307. 32204974 10.1016/j.clnu.2020.02.037

[35] Yajnik CS , Deshpande SS , Lubree HG , et al. Vitamin B12 deficiency and hyperhomocysteinemia in rural and urban Indians. J Assoc Physicians India. 2006;54:775‐782. 17214273

[36] Kang D , Yun JS , Ko SH , et al. Higher prevalence of metformin‐induced vitamin B12 deficiency in sulfonylurea combination compared with insulin combination in patients with type 2 diabetes: a cross‐sectional study. PLoS One. 2014;9:e109878. 25299054 10.1371/journal.pone.0109878 PMC4192538

[37] Out M , Kooy A , Lehert P , Schalkwijk CA , Stehouwer CDA . Long‐term treatment with metformin in type 2 diabetes and methylmalonic acid: post hoc analysis of a randomized controlled 4.3 year trial. J Diabetes Complications. 2018;32:171‐178. 29174300 10.1016/j.jdiacomp.2017.11.001

