# Supplementary material for: The effects of daily dose and treatment duration of metformin on the prevalence of vitamin B12 deficiency and peripheral neuropathy in Chinese patients with type 2 diabetes mellitus: A multicenter cross‐sectional study
Source: J Diabetes. 2023 Jun 13;15(9):765–76. doi: 10.1111/1753-0407.13428 (PMC10509512; doi:10.1111/1753-0407.13428)
Supplement: Supplementary file 1 — Appendix S1. [file JDB-15-765-s001.docx]

**Exclusion criteria:**

1、Other than type 2 diabetes;

2、The dose of metformin was continuously adjusted (or discontinued) for>7 days within 6 months before screening;

3. Any episodes of acute diabetic complications within 6 months before screening, including diabetes ketoacidosis with or without coma, or lactic acidosis;

4. Severe trauma that may affect the control of blood glucose within 1 month before screening;

5. Viral, bacterial, fungal, parasitic, or mycobacterial infections requiring systemic treatment within one month before screening;

6. Repeated episodes of severe hypoglycemia judged by the investigator;

7. Severe chronic comorbidites, such as myocardial infarction, cerebrovascular accident, congestive heart failure, blindness caused by diabetes retinopathy, renal failure and severe foot ulcer;

8. Uncontrolled endocrine diseases other than type 2 diabetes;

9. Diseases of the blood system (such as aplastic anemia, myelodysplastic syndrome, etc.), or any disease that causes hemolysis or red blood cell instability (such as malaria, hemolytic anemia, etc.);

10. Stomach and intestinal diseases related to significant digestive and absorption disorders that may affect drug absorption;

11. A history of gastrointestinal reactions to antidiabetic treatments (such as GLP-1 receptor agonists );

12. A history of liver diseases such as acute or chronic hepatitis, with obvious clinical symptoms or signs;

13. A history of acute or chronic kidney disease;

14. A history of chronic obstructive pulmonary disease, uterine fibroids, and other diseases with obvious clinical symptoms such as moderate to severe anemia;

15. A history of malignant tumor(s) within 5 years before screening (except for specific cancers that have been surgically removed and completely cured, such as skin basal cell carcinoma, or cervical carcinoma in situ), or potential malignant tumors are currently being evaluated;

16. Central nervous system disease or spinal cord injury (such as cervical and lumbar lesions, cerebral infarction, Guillain Barre syndrome, chronic inflammatory demyelinating polyneuropathy, etc.);

17. Other nervous system diseases that may affect the evaluation of diabetes peripheral neuropathy according to the evaluation from investigators;

18. A history of drug abuse or daily drinking>2 standard doses within 6 months before screening (one standard dose was 285ml beer, 375ml light beer, 100ml red wine or 30ml Baijiu, each containing about 10g alcohol), or other persistent conditions, which would reduce their compliance with the research procedure according to the evaluation from investigators;

19. Vegetarians;

20. A history of vitamin B12 treatment;

21. A history of folic acid treatment within 6 months before screening;

22. A history of surgical treatment for colorectal, bladder, or prostate, etc;

23. subject participating in an intervention clinical study.

24. Female who is pregnant, breast-feeding.
